# Some Remarks on Lacerations of the Perineum; a Plea for More Thoroughness in the Prevention, Also for the Immediate Repair; the Author’s Technique*Read before the Eighth District Medical Society, October 8, 1908, at Victoria, Texas.

**Published:** 1909-04

**Authors:** Addison L. Lincecum

**Affiliations:** Louise, Texas


For Texas Medical Journal.

### Some Remarks on Lacerations of the Perineum; A Plea for More Thoroughness in the Prevention, Also for the Immediate Repair; The Author's Technique.*

BY ADDISON L. LINCECUM, M. D., LOUISE, TEXAS.

It has been the rule among country obstetricians, in the past, to neglect the repair of recent lacerations of the perineum; usually treating them with a dressing of carbolized olive oil or some mild antiseptic, and allowing them to heal by granulation, thereby resulting in a massive cicatrix, leaving the vagina without a floor to support the uterus and appendages, resulting in prolapsus, procedencia, cystocele, rectocele, or the various malpositions of the pelvic viscera.

Such negligence, in the writer's opinion, is criminal, and my earnest plea is for a more thorough attempt at the prevention of lacerations of the perineum, and when they do occur for their immediate repair.

### PREVENTION.

The first step at prevention is to educate the laity. Teach them that an uneducated midwife is not competent to accouche a woman. Teach them that when a wife becomes pregnant (especially if she is a primipara), she should be placed in the hands of a competent physician for observation during the entire period of gestation and accouchement. It is the duty of the obstetrician assuming this trust to instruct her about her clothing, hygiene, exercise, diet, etc., and at the time of her accouchement she will be in the prime condition, both physically and mentally. Then he may expect an ideal accouchement, and will not be placed in the position that we have been in, many times, by being called, posthaste, to see a woman who is in labor, or has just been delivered and lacerated by a filthy negro midwife, or some neighbor woman who is equally as ignorant. Upon our arrival, we find conditions bad, the husband has no money, we hastily deliver the placenta, finish up as quickly as possible, and leave the laceration to repair itself, and run the risk of infection through an open wound, followed by sepsis and death to a woman. Why? First, because the laity are not educated

*Read before the Eighth District Medical Society, October 8, 1908, at Victoria, Texas.

to the importance of scientific care. Second, commercialism has the upperhand of the duty we owe to womankind for our mother's sake, if nothing else. The second method of preventing, or reducing perineal lacerations to the minimum, lies entirely in the hands of the accoucheur.

There are two common causes of lacerations. One is instrumental traumatism, which occurs in about 50 per cent of the cases so delivered. The other is a full rectum and bladder, with rigidity of the perineal muscles accompanied by powerful contractions and rapid descent of the child. When this condition exists, the writer, under strict aseptic conditions, introduces the fingers of the right hand into the vagina and uses gentle but firm traction against the perineum, stretching it outward like the small boy does a "nigger-shooter" rubber, until we have forcibly dilated it until the head will pass with little or no resistance. When the head becomes engaged against the perineum we place the palm of one hand against the outer surface of the perineum, and press firmly downward, allowing the head to pass slowly, but with perfect ease; then during the period of rest that follows the delivery of the head we deliver the shoulder and arm that engages the pubic bone, as the shoulder that engages the perineum is the real weapon of laceration. If the superior shoulder is not delivered the next uterine contraction forces the inferior shoulder outward and it nearly rips the perineum open. Now, if the superior shoulder is delivered before this occurs, the inferior shoulder assumes an angle of about 45 degrees, and easily and harmlessly passes the perineum, but, should laceration occur, my plea is for immediate repair. The technique described below has been successful in my hands in 21 out of 24 cases; i. e., primary union in 21 cases, infection from filthy nurses in 2 cases and gonorrhea in 1 case.

Before describing the operative technique, let us discuss the four factors which so often prevent primary union in cases repaired immediately after delivery:

1. Pressure necrosis, with sloughing.
2. Leakage of the uterine and vaginal discharge into the newly approximated wound.
3. Infection and suppuration, especially if gonorrhea exists.
4. Lack of thoroughness in the preparation of our field of operation and bad suture material used therein.

I have made here some crude drawing to endeavor to make plain each step in the operation.

TECHNIQUE.

Immediately after the placenta is delivered the patient is placed on a Kelly pad in the dorsal position, with the limbs strapped up or held up by an assistant, the vulva is shaved and scrubbed, then rinsed; the vagina is flushed out with hot saline solution, then plugged with sterile gauze to prevent the uterine discharges from flowing into our field of operation. The lacerated surfaces are now separated and the ragged blackened tissue which looks necrotic is removed with the scissors. If left, all this tissue will slough and prevent union, and may absorb and cause sapremia. The surfaces being now ready for approximation, we take a large curved needle threaded with silkworm gut and pass from two to six through-and-through sutures, the number varying according to the degree of the laceration. We begin by putting in the bottom suture first, taking a bight of three-eighths to one-fourth of an inch, using the index finger of the left hand as a guide to be sure that the needle does not pass through the mucous membrane into the vagina, also to keep from puncturing the rectum; pass the suture through the entire muscular structure and make an exit the same distance from the laceration as the point of entrance, except on the opposite side. We insert sufficient number of these sutures to perfectly approximate the surface (see Fig. 2). (Do not tie these suture until the last step is reached.)

The next step in the operation is to take a small curved needle, threaded with No. 1, plain sterile catgut; beginning at the vaginal end of the laceration put in a continuous side-to-side suture, continuing to the outer margin of it, tying just subcutaneously, place layer above layer until the laceration is obliterated and there is no possible chance of leakage into the wound. (See Fig. 2.)

The silkworm gut sutures are merely a protecting anchor, against accident. They are now tied, and the ends placed into a hollow shot or a piece of rubber tubing to prevent irritation. (See Fig. 3.)

The gauze is now removed from the vagina, and a sterile gauze pad and a T bandage is applied as a dressing.

The nurse is instructed to keep the sutured surface clean and dry, and we personally irrigate the vagina each day.

We instruct the nurse that when the patient wants to void the urine, to place the Kelly pad on the side of the bed with the sleeve hanging into a vessel; roll the patient on her belly and let her void it, and thus save the freshly operated field from contamination.

The bowels are kept open, and the patient is bathed and dried after each stool.

On the fourteenth to eighteenth day the silkworm gut is removed, one or two sutures at a sitting, the catgut having been absorbed.

RECAPITULATION.

1. By educating the laity to the importance of scientific care of these cases we can reduce the predisposing causes of lacerations to the minimum, and by careful assistance to the parturient woman we can reduce the occurrence also.

2. By rigid asepsis, and thorough preparation of the surfaces before approximation, and by perfect care in executing the work, using only the best suture material, we render the possibility of interference from infection and leakage quite small.

3. By the very best of after care being scrupulously clean we expect only good results.

